# Skeletal muscle-directed gene therapy: hijacking the fusogenic properties of muscle cells

**DOI:** 10.1038/s41392-023-01584-4

**Published:** 2023-09-13

**Authors:** Hildegard Büning, Michael Morgan, Axel Schambach

**Affiliations:** 1 Institute of Experimental Hematology, Hannover Medical School, Hannover, 30625 Germany; 2 REBIRTH Research Center for Translational Regenerative Medicine, Hannover Medical School, Hannover, 30625 Germany; 3 Division of Hematology/Oncology, Boston Children’s Hospital, Harvard Medical School, Boston, MA 02115 USA

**Keywords:** Molecular medicine, Translational research

A recent study published in *Cell* by the Millay lab reports on an elegant strategy to expand the repertoire of lentiviral (LV) vectors from being the main delivery tool for ex vivo gene therapy to an in vivo applicable viral vector that specifically transduces skeletal muscle.^1^ This very significant advance may result in novel therapeutics for patients suffering from various skeletal muscle diseases.

With 40% of the total body weight, skeletal muscle is one of the largest organs of our body.^2^ As many monogenetic diseases affect skeletal muscle function, such as muscular dystrophies (e.g., Duchenne Muscular Dystrophy) and myopathies, musculature is a main target of gene therapy. Moreover, skeletal muscle is considered as a promising production hub for recombinant proteins to be continuously secreted into the blood stream, such as blood clotting factors or neutralizing antibodies.^3^ Skeletal muscles are unique as they are a syncytium of individual muscle cells that are fused into a multinuclear muscle fiber. By deciphering the process of syncytium formation during muscle cell development and muscle regeneration following injury, Myomarker and Myomerger (also Myomixer/Minion) were identified as the responsible fusogens.^4^ Since the fusion process in syncytium formation reminded the authors of the first step in cell infection by enveloped viruses, i.e. the fusion of the viral envelope with a cell membrane at the surface or at the endosomal state, they investigated whether the muscle-cell-specific fusogens Myomaker and/or Myomerger could be used to direct cell transduction of enveloped viral vectors.^1^ Indeed, they impressively showed that the vesicular stomatitis virus (VSV) G protein (VSV-G), a viral fusogen, that is used to mediate cell transduction can be replaced by Myomaker (Mymk) and Myomerger (Mymg). VSV-G depleted, Mymk and Mymg pseudotyped VSV particles transduced primary mouse myotubes, while non-proliferating myoblasts and fibroblasts appeared refractory, a tropism in line with the expression profile of Myomaker, one of the two muscle-specific fusogens shown by Hindi and colleagues to be required on the target cell to mediate transduction by Mymk+Mymg pseudotyped particles. Importantly, pseudotyping via Mymk+Mymg was not restricted to VSV. In contrast, extracellular vesicles, which are clearly non-viral structures, as well as LV vectors could be redirected towards skeletal muscle cells. Mymk and Mymg pseudotyped LV vectors, termed Mymk+Mymg-LV, were independent of the VSV-G protein, which commonly renders pseudotyped LV vectors sensitive to complement inactivation.

As muscle-directed gene therapy strategies are commonly applied as in vivo approaches, as a next milestone, Mymk+Mymg-LV was explored in mice initially by local and then by retroorbital administration. These comprehensive investigations confirmed the tropism of Mymk+Mymg-LV for skeletal muscle. Successful transduction required muscle injury or a hypertropic stimulus, a requirement explained by the expression profile of Myomaker and Myomerger. Specifically, muscle-specific fusogens are absent in mature myofibers and quiescent satellite cells, the muscle stem cells, but are expressed by activated muscle satellite cells for example following muscle injury. Interestingly, while Myomaker and Myomerger need to be present on the target cell membrane during muscle development or regeneration,^4^ presence of Myomaker on the target cell—as mentioned above–appeared to be sufficient to mediate Mymk+Mymg-LV in vivo transduction.^1^ The strict dependency of Mymk+Mymg-LV on the presence of this fusogen for cell transduction combined with the timely and spatially restricted natural expression of Myomaker confers the system with a unique specificity: (1) no transduction of liver, a common off-target of intravenously applied viral and non-viral vector systems, including lipid nanoparticles (LNP) or adeno-associated virus (AAV) vectors, (2) no transduction of other non-muscle tissue like kidney or spleen and (3) no transduction of non-skeletal muscle tissue like heart. In contrast, all skeletal muscles, including the diaphragm, were transduced. This also holds true for diseased conditions, as shown in a dystrophic mouse model.

However, efficient transduction of skeletal muscle cells is dependent—as mentioned—on activated myogenic progenitors, which then fuse to muscle fibers as transduced satellite cells. Due to this mechanism, the transduction efficiency of Mymk+Mymg-LV for skeletal muscle is significantly lower than efficacies reported by a single administration of AAV vectors.^1,3^ To increase efficacy, multiple subsequent administrations are possible, with the potential to yield up to 78% transgene-expressing myofibers as reported in this study. The latter efficacies were reported for a therapeutically relevant transgene, microdystrophin, which is even more impressive. The option of repeated administration deserves special recognition as redosing with viral vectors is commonly not possible due to the induction of neutralizing antibodies.

Thus, this novel approach – developed by the Millay lab - addresses one of the main challenges to gene and cell therapies, namely, a cell-type selective in vivo delivery of a therapy to the medically relevant tissue following intravenous administration. The specificity of the approach is potentially clinically applicable and relevant for a variety of muscle diseases.

**Fig. 1:**
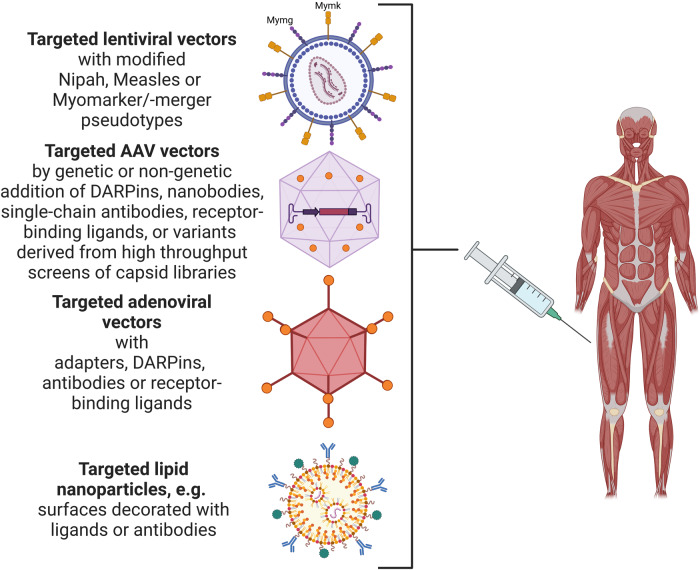
Targeting principles for delivery of gene therapies. Hindi et al. report on a novel strategy to selective target skeletal muscle cells in vivo by transducing activated stellate cells with lentiviral vectors equipped with the muscle-specific fusogens Mymg and Mymk. This adds to the available strategies of changing the tropism of lentiviral vectors. Current strategies employ pseudotyping with Nipah virus or Measles virus-derived surface glycoproteins that are blinded for their natural tropism and equipped with new targeting specificities, e.g. peptides, antibodies, DARPins. Similarly, non-enveloped viral vectors such as adeno-associated viruses or adenoviral vectors can be equipped with DARPins, nanobodies, single-chain antibodies, or receptor-binding ligands either genetically or non-genetically (modified sites for specific targeting are indicated as orange circles). The key to obtaining a re-directed tropism is the combination of blinding the particle for the natural receptor usage with insertion of targeting moieties. Cell surface targeting technologies have more recently also been expanded to lipid nanoparticles to broaden their in vivo usage. Targeted delivery strategies can be exploited for the expression of transgenes and tools for RNA interference as well as gene/base editing. Figure created with BioRender.com

Initial attempts to change the tropism of LV vectors relied on the exchange of VSV-G with modified retargeted glycoproteins derived from Nipah and Measles viral envelopes (Fig. 1). Cell surface targeting technologies have also been developed for non-enveloped viral vectors, such as vectors derived from adenovirus (AdV) or AAV.^3,5^ They have been explored to enable cell transduction of cell types refractory to transduction of a given vector due to lack of cognate receptors. With regard to systemic in vivo gene therapy, the field has emphasized tropism redirection rather than broadening transduction profiles, i.e. to restrict cell transduction towards target cells of a given treatment approach. To reach this goal, particles have to be blinded for their natural receptor interaction and outfitted with a new specificity through the introduction of receptor-binding ligands or protein-based adaptor molecules, such as Designed Ankyrin Repeat Proteins (DARPin), nanobodies or single-chain antibodies (Fig. 1). The key to specificity is the availability of cell surface molecules that are indeed target cell-specific. As such, the strategy of cell surface targeting of enveloped vectors reported here is an impressive example of what is possible. Translation toward non-enveloped vectors is likely a challenging endeavor. However, exploring these new skeletal muscle-directed LV vectors in combination with another delivery system such as AAV vectors to empower muscle gene therapy approaches appears—as already discussed by the authors—as a promising avenue to follow.
